# Supplementary material for: Tailored haemodynamic response function increases detection power of fMRI in awake dogs (Canis familiaris)
Source: Neuroimage. Author manuscript; Available in PMC 2024 Aug 7. (PMC7616344; doi:10.1016/j.neuroimage.2020.117414)
Supplement: Supplementary material [file EMS196378-supplement-Supplementary_material.docx]

**Supplementary material**

# Monitor eye tracker camera

| 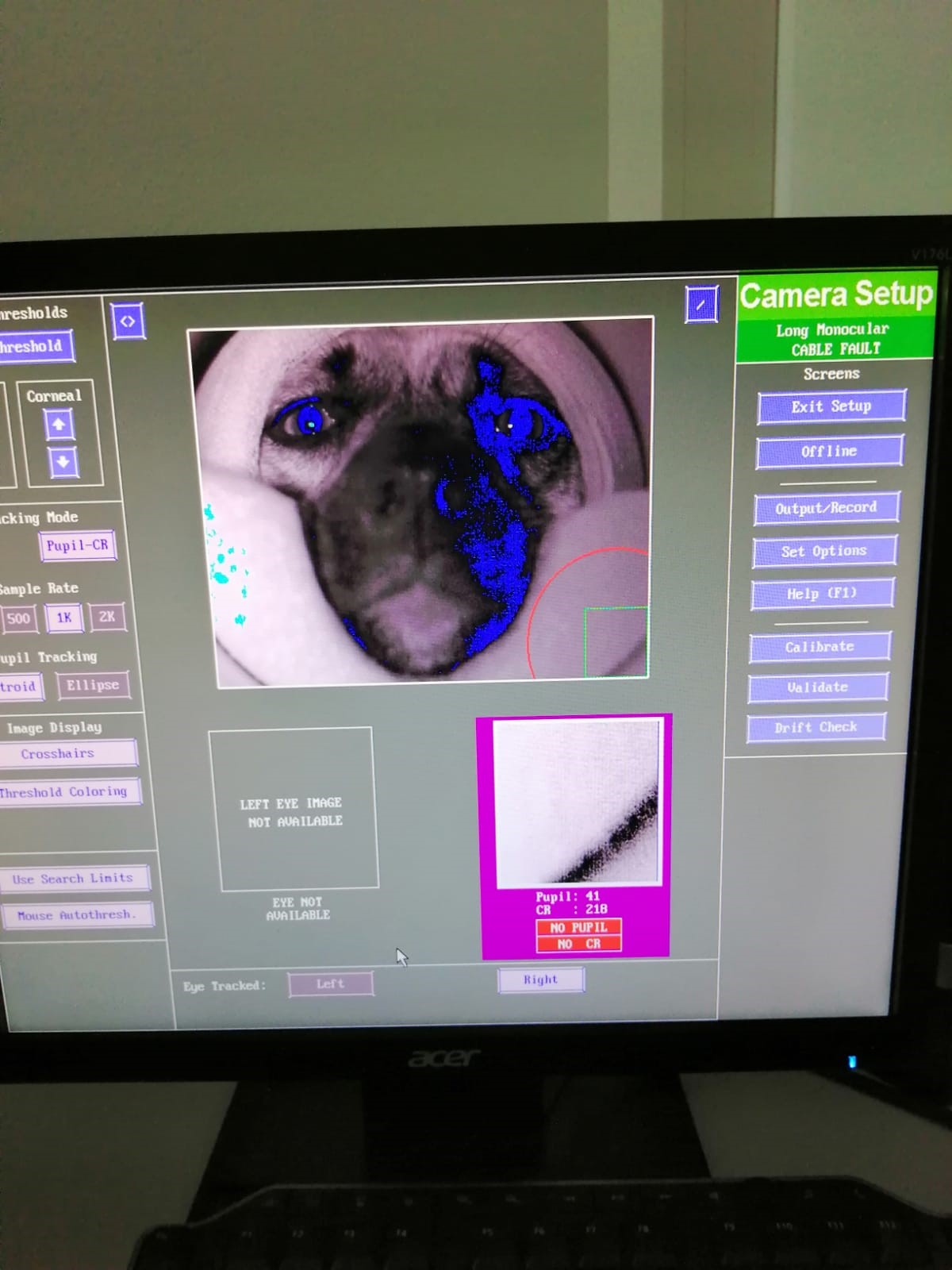 |
| --- |
| ***Figure S1***. Live monitoring of a dog during data collection. The live recordings of the dogs using the camera of the eye tracker (Eyelink 1000 Plus, SR Research, Ontario, Canada) allowed us to monitor their attention, looking behaviour, and overall motion. |

# Flipping experiment 1 and 2

In the present study, we estimated a tailored dog haemodynamic response function (HRF) based on a block design (dog HRF flickering checkerboard experiment; see 2.5 fMRI data analysis), and then validated the results with an event-related design (independent data, face processing experiment). The majority of studies investigating the shape of the BOLD signal time course employed an event-related design; but see Shan et al., 2014). In order to further validate the dog HRF based on the flickering checkerboard experiment, we also estimated a dog HRF based on the face processing experiment (dog HRF face processing experiment) and calculated the model fit for both data sets (see 2.5.1 Exploration and estimation analysis, step 2 + 3 and 2.5.2 Validation analysis, step 2 for description of estimation procedure and model fit calculation).

**Model fits: FIR data flickering checkerboard experiment**

Human HRF: *R^2^* = 0.35 ± 0.20

Dog HRF flickering checkerboard experiment: *R^2^*= 0.64 ± 0.21

Dog HRF face processing experiment (if we flipped experiments): *R^2^*= 0.61 ± 0.21

Both, the dog HRF estimated based on the average BOLD signal from the flickering checkerboard experiment and the dog HRF based on the average BOLD signal extracted from the face processing experiment resulted in an increased model fit (i.e., *R^2^*-statistics) compared to the human HRF. The dog HRF models resulted in almost numerically identical results. Thus, both dog HRF estimates fitted the average V1 BOLD signal from the flickering checkerboard experiment with a comparable accuracy, and both dog HRF estimates modelled an earlier peak as compared to the human HRF (see supplemental figure S2A).

**Model fits: FIR data face processing experiment (run 1)**

Human HRF: *R^2^* = +0.06 ± 0.11

Dog HRF flickering checkerboard experiment: *R^2^* = +0.50 ± 0.31

Dog HRF face processing experiment: *R ^2^*= 0.54 ± 0.32

**Model fits: Data face processing experiment (run 2)**

Human HRF: *R^2^* = 0.15 ± 0.22

Dog HRF flickering checkerboard experiment: *R^2^* = 0.44 ± 0.31

Dog HRF face processing experiment: *R^2^* = 0.44 ± 0.34

For both experimental runs of the face processing experiment, the two dog HRF estimates resulted in an increased model fit modelling an earlier peak (see supplemental figure S2B) compared to the human HRF. Again, the dog HRFs resulted in similar model fits.

In summary, our results remained stable when analyses of the experiments were flipped, i.e., when we used the average BOLD signal from the face processing experiment (event-related design) instead of the flickering checkerboard experiment (block design) to estimate the dog HRF.

| ­ 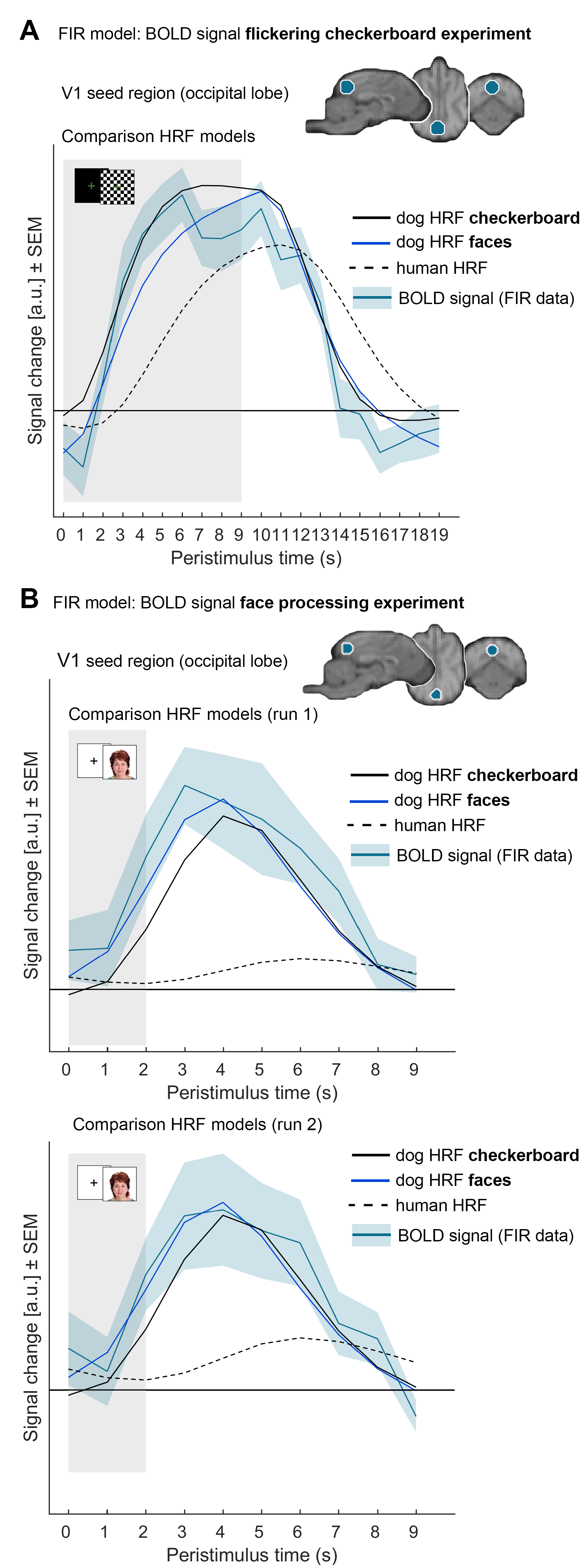 |
| --- |
| ***Figure S2***. Averaged BOLD signal time courses based on blocked (flickering checkerboard, experiment 1) and event-related designs (face processing, experiment 2) resulted in an earlier peak than predicted by the human HRF. Dog HRF estimations based on both designs yielded similar results. We calculated finite impulse response (FIR) models and extracted the average BOLD signal time courses from the primary visual cortex (V1) for (**A**) the flickering checkerboard experiment (exploration and estimation analysis, step 5) and (**B**) the face processing experiment (both runs separately; validation analysis, step 4). Then, to validate the accuracy of the tailored dog HRF (dog HRF checkerboard), we calculated another dog HRF based on the face processing experiment (dog HRF faces). A visual comparison revealed similar results: both dog HRF estimates predicted an earlier peak than the human HRF. To display the fit of the HRF models for both experiments, the dog HRF estimates and the human HRF are plotted in addition to the extracted BOLD signal time course. For illustration purposes, the dog and human HRFs were scaled by the parameter estimates (arbitrary units, a.u.) from the respective GLMs. SEM, standard error of the mean. |

# References

Friston, K. J., Jezzard, P., & Turner, R. (1994). Analysis of functional MRI time-series. *Human Brain Mapping*, *1*(2), 153–171. https://doi.org/10.1002/hbm.460010207

Handwerker, D. A., Ollinger, J. M., & D’Esposito, M. (2004). Variation of BOLD hemodynamic responses across subjects and brain regions and their effects on statistical analyses. *NeuroImage*, *21*(4), 1639–1651. https://doi.org/10.1016/j.neuroimage.2003.11.029

Lindquist, M. A., Meng Loh, J., Atlas, L. Y., & Wager, T. D. (2009). Modeling the hemodynamic response function in fMRI: Efficiency, bias and mis-modeling. *NeuroImage*, *45*(1). https://doi.org/10.1016/j.neuroimage.2008.10.065

Shan, Z. Y., Wright, M. J., Thompson, P. M., McMahon, K. L., Blokland, G. G. A. M., De Zubicaray, G. I., … Reutens, D. C. (2014). Modeling of the hemodynamic responses in block design fMRI studies. *Journal of Cerebral Blood Flow and Metabolism*, *34*(2), 316–324. https://doi.org/10.1038/jcbfm.2013.200
